# Accelerated Infliximab Infusion Safety and Tolerability Is Non-inferior to Standard Infusion Protocol in Inflammatory Bowel Disease Patients: A Randomized Controlled Study

**DOI:** 10.1093/crocol/otad022

**Published:** 2023-05-03

**Authors:** Suha Abushamma, Ted Walker, Kevin Garza, Ling Chen, Darren Nix, Chien-Huan Chen

**Affiliations:** Division of Gastroenterology, John T. Milliken Department of Medicine, Washington University School of Medicine, Saint Louis, Missouri, USA; Division of Gastroenterology, John T. Milliken Department of Medicine, Washington University School of Medicine, Saint Louis, Missouri, USA; Division of General Medicine, John T. Milliken Department of Medicine, Washington University School of Medicine, St. Louis, Missouri, USA; Division of Biostatistics, Washington University School of Medicine, Saint Louis, Missouri, USA; Division of Gastroenterology, John T. Milliken Department of Medicine, Washington University School of Medicine, Saint Louis, Missouri, USA; Division of Gastroenterology, John T. Milliken Department of Medicine, Washington University School of Medicine, Saint Louis, Missouri, USA

**Keywords:** infliximab, inflammatory bowel disease, accelerated infusion

## Abstract

**Background and Aim:**

Infliximab is typically given over an infusion time of 2 hours, leading to a significant burden in inflammatory bowel disease (IBD) patients. We aimed to determine the safety and cost-effectiveness of an accelerated infliximab infusion of 1 hour, compared with the standard 2-hour infusion.

**Methods:**

Open-label randomized trial where IBD patients receiving maintenance infliximab infusions were randomly assigned to 1- and 2-hour infusion groups, corresponding to study and control groups, respectively. The primary outcome was the rate of infusion reactions. Secondary outcomes were assessment of the effect of premedications and immunomodulators on the rate of infusion reactions, and cost-effectiveness analysis. The cost-effectiveness analysis was based on direct nursing costs for the infusion time, indirect infusion center costs, and cost of productivity loss for patients. This trial is registered with ClinicalTrials.gov, NCT05340764.

**Results:**

From November 2020 to November 2021, 96 patients were randomly assigned: 51 (53%) to the 1-hour infusion group and 45 (47%) to the 2-hour infusion group. Over a median time of 1 year, 309 infusions were administered in the control group, and 376 in the study group. Fifty-seven (18%) infusions in the control group and 45 (12%) infusions in the study group experienced an infusion reaction. The only infusion reaction was asymptomatic hypotension not requiring infusion discontinuation. No other infusion reactions (mild or moderate/severe) were seen. Diphenhydramine was associated with an increased rate of infusion reactions (OR 2.04 [95% CI 1.18–3.52], *P* = .01). The average costs were estimated to reduce by 37% in the accelerated infusion group.

**Conclusions:**

Accelerated 1-hour infusions are non-inferior in safety and superior in cost-effectiveness compared with standard 2-hour infusions in IBD patients receiving maintenance infliximab infusions.

**Trial Identification Number:**

Registered with ClinicalTrials.gov, NCT05340764.

## Introduction

Inflammatory bowel disease (IBD) is a chronic illness with a remitting and relapsing course often diagnosed at a young age, with 2 discrete entities: ulcerative colitis and Crohn’s disease.^1^ In the United States, approximately 0.4%–0.6% of the population is affected by IBD,^2^ with increasing prevalence in recent years and significant associated healthcare resource utilization and costs.^3,4^ Tumor necrosis factor (TNF) alpha is a proinflammatory cytokine that was discovered in the 1970s^5^ and is a major factor implicated in the pathogenesis of IBD.^6^ TNF inhibitor therapy was therefore trialed in IBD patients with high success rates,^7–9^ which led to the FDA’s approval of infliximab, the first TNF inhibitor, in 1998.^10^

Since infliximab is a chimeric monoclonal antibody given through intravenous infusion, the potential for acute infusion reactions has been vigilantly assessed and reported. Earlier studies have reported a 5%–23% incidence rate for infusion reactions^11–13^; however, more recent studies suggest that the rate is much lower, at 1.3%–3.5%.^14–17^ Premedications including acetaminophen, antihistamines, and corticosteroids have been used to mitigate the risk of reactions, although their effectiveness has not been established.^16,18^ On the other hand, concomitant immunomodulators are used to decrease the risk of antibody formation to infliximab, and have been shown to reduce the risk of infusion reactions.^19,20^ Given the concern for infusion reactions, infliximab is currently approved by the FDA to be given over 2 hours or more.^21^ The typical interval for infusions is every 8 weeks but is at times reduced to every 6 or even 4 weeks in patients with IBD. This infusion time represents a significant inconvenience to patients who receive regular maintenance infusions, and is associated with lower patient satisfaction rates.^22,23^

As a result, there has been increasing interest in an accelerated infliximab infusion time in recent years. Multiple studies outside of the United States have demonstrated that a shortened infusion time to 1 hour and even 30 minutes is safe and tolerable with similar rates of infusion reactions compared with an infusion time of 2 hours.^24–30^ It has also been shown that reducing infusion times leads to cost savings and increased patient satisfaction.^30–32^ A recent large retrospective study conducted in the United States again showed that a shortened infusion time of 1 hour is safe and well tolerated.^33^ While previous studies provide evidence that 1-hour infusions appear to be safe, selection bias and confounding factors could not be completely eliminated in retrospective studies. To our knowledge, there are no randomized trials comparing standard and accelerated infliximab infusions. Hence, in this study we aimed to compare the infusion reaction rate of an accelerated infliximab infusion time of 1 hour, to the standard 2-hour infusion in a contemporary IBD practice using a randomized controlled trial.

## Materials and Methods

### Study Design and Participants

This single center, open-label, randomized clinical trial done at a tertiary IBD center in the United States, was designed to determine the safety of 1-hour infliximab infusions compared with the standard 2-hour infusions in IBD patients. The protocol was approved by the Institutional Review Board (IRB), and the trial was executed in accordance with the study protocol. It was registered with ClinicalTrials.gov under identifier NCT05340764.

Participants were recruited from 2020 to 2021 and followed throughout the study after enrollment. Patients aged 18 years or older with an established diagnosis of IBD, receiving infliximab at one of our infusions centers and have tolerated the 3 induction doses and current maintenance dosing were eligible for inclusion in the study. Patients were excluded if they were receiving infliximab exclusively for a non-IBD indication, had a history of moderate or severe infusion reactions to infliximab (or biosimilar), had known antidrug antibodies to infliximab, had an interval greater than 13 weeks from prior dose of infliximab, were receiving an additional infusion concomitant with infliximab (eg, IV iron), or declined to participate in the trial. The electronic medical record was queried for potentially eligible patients using the Slicer-Dicer function of Epic. Study coordinators then reviewed patients’ charts to determine eligibility. Eligible patients were contacted and invited to participate consecutively by phone by one of the study coordinators.

### Randomization and Masking

Informed consent was obtained from all patients prior to randomization in the study. Patients were randomized (1:1) into the 1-hour infusion (study) and 2-hour infusion (control) groups using an independent computerized randomization system. Patients and researchers were not able to be masked to group allocation after randomization.

### Study Procedures

**Figure 1.**
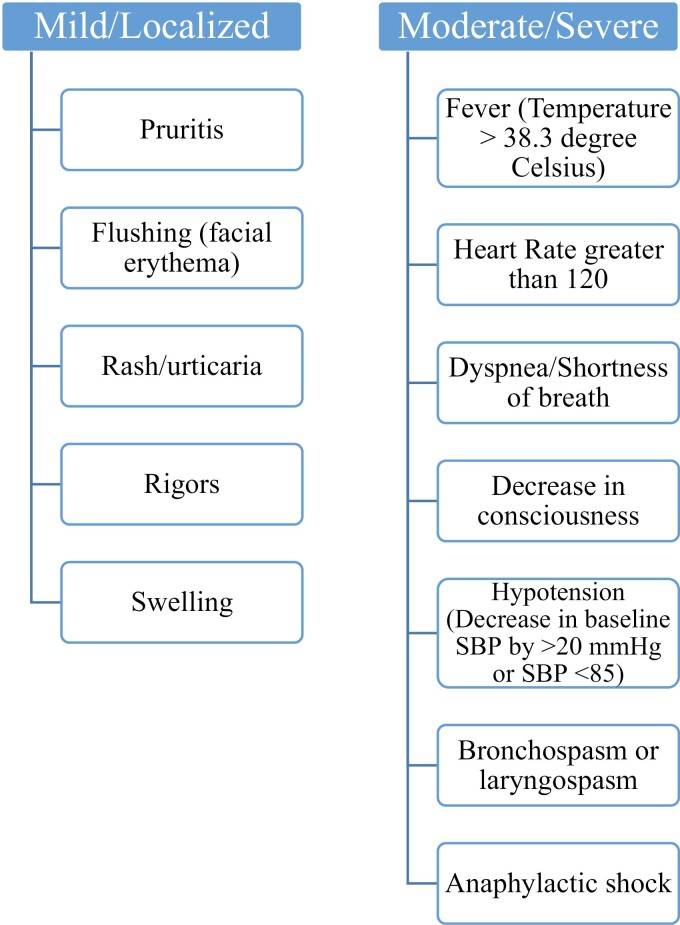
Infliximab acute infusion reactions.

Patients were randomized to study or control groups and monitored for infusion reactions during or immediately after completion of the infusion. Infusion reactions were divided into mild/localized and moderate/severe reactions as illustrated in Figure 1. Treatment for any infusion reaction was carried out according to our center’s standard algorithm. All infusion doses as well as infusion intervals were eligible for inclusion in the study. Drug dosing and intervals were determined by the prescribing physician and were documented. Additional information including IBD type, demographics, premedication use, and concomitant immunomodulatory therapy was recorded as well.

### Outcomes

The primary outcome was the rate of infusion reactions in the standard 2-hour control group compared with the accelerated 1-hour study group assessed through study completion.

Secondary outcomes were assessment of the effect of premedications and concomitant immunomodulators on the rate of infusion reactions, and cost savings analysis of the accelerated infliximab infusion time. The cost savings analysis was based on direct nursing costs for the infusion time and indirect infusion center costs, including nursing costs for administering the infusion, monitoring of patients, and ancillary paperwork. This was calculated based on the average registered nurse salary and infusion center chair occupancy cost. In addition, cost of productivity loss for patients during the infusion time and time spent traveling to the infusion center was calculated. We also used the chair occupancy of the infusion center to calculate the change of revenue based on different infusion duration.

### Sample Size Calculation and Statistical Analysis

Our sample size calculation was based on an infusion reaction rate of 3%, as suggested by recent studies.^14–17^ In order to show a non-inferiority of the accelerated infusion time assuming an infusion reaction rate of 3% while allowing for a 4% non-inferiority limit, a total of 538 infusions would be needed. This would provide a power of 80% and significant alpha of 0.05.

Results were expressed as mean (SD) or median (interquartile range—IQR) for continuous variables and frequency (percentage) for categorical variables. Continuous data with a normal distribution were compared using the Student *t*-test. Categorical data were analyzed using the χ^2^ test. Farrington–Manning score test based on risk difference was performed to test for non-inferiority of infusion reaction rates using a non-inferiority margin of 4%. A test-based 2-sided 90% CI for the rate difference was reported. To test for association between infusion reactions and concomitant immunosuppression as well as premedications, a generalized estimating equation modeling was used and odds ratios with 95% CI were reported. A significance level of 0.05 was used for all statistical tests. Data were analyzed with SAS version 9.4 (SAS Inc).

## Results

### Patient Demographics

**Figure 2.**
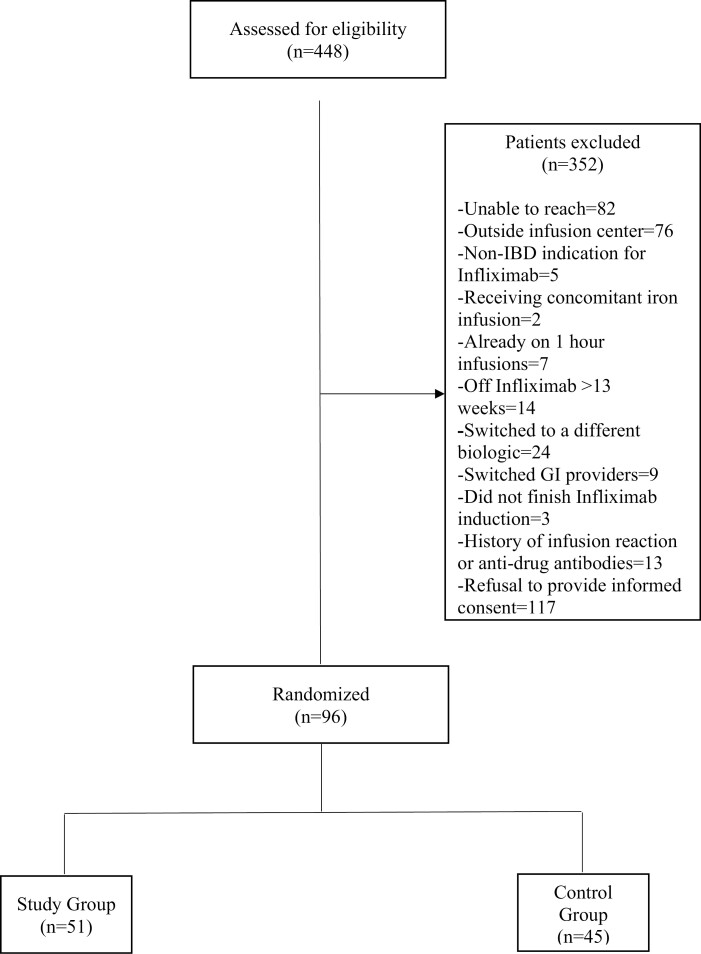
Patient flowchart.

Between November 2020 and November 2021, 448 patients were assessed for eligibility, of whom 352 (78%) were excluded primarily due to inability to reach the patient, infusions being received at an outside institution, or patient refusal (Figure 2). Patients were not asked for an explanation for their refusal, however refusal was not due to history of infusion reactions, receiving concomitant infusions, or any of the other criteria for exclusion, as these were recorded separately. Ninety-six (22%) patients were randomly assigned: 51 (53%) to the 1-hour infusion study group and 45 (47%) to the 2-hour infusion control group. Excluded patients who refused to be admitted to the study and patients receiving infusions at outside institutions (*n* = 193) were compared with patients included in the study, and no significant differences were found in their demographics, IBD classification, and infliximab data (Supplementary Table S1).

Mean age was 47 (SD 15.5) in both groups in the trial. About 3 quarters of patients had Crohn’s disease and half were on concomitant immunomodulators. There were no differences in patient demographics between the 2 groups (Table 1).

**Table 1.** Patient demographics.

	2-h infusion group (*n* = 45)	1-h infusion group (*n* = 51)
Age	47.18 ± 15.8	46.94 ± 15.4
Gender		
Male	23 (51)	28 (55)
Female	22 (49)	23 (45)
IBD type		
UC	11 (24)	10 (20)
CD	34 (76)	41 (80)
Concomitant immunomodulator		
6-MP	8 (18)	5 (10)
AZA	9 (20)	11 (21)
MTX	5 (11)	10 (20)
None	23 (51)	25 (49)

Data are expressed in median (IQR), mean ± SD, or *n* (%). Abbreviations: 6-MP, 6-mercaptopurine; AZA, azathioprine; CD, Crohn’s disease; IBD, inflammatory bowel disease; MTX, methotrexate; UC, ulcerative colitis.

### Infliximab Data

Almost half of the patients were on infliximab 10 mg kg^−1^ and most were on an every 8 week infusion interval (Table 2). Patients were on 10 mg kg^−1^ dosing due to low trough levels or significant diseases that necessitate dose escalation, such as perianal complication, penetrating disease, or extra-intestinal manifestations (pyoderma gangrenosum, erythema nodosum, psoriasis, ankylosing spondylitis, and arthralgias). The originator infliximab (REMICADE) was the predominant infliximab given, while up to a quarter of patients had received biosimilar or both originator and biosimilar, as dictated by insurance. Seven patients in the control group and 14 patients in the study group switched from originator to biosimilar or vice versa during their study participation. There were no differences in infliximab characteristics between the 2 groups (Table 2).

**Table 2.** Infliximab data.

	2-h infusion group (*n* = 45)	1-h infusion group (*n* = 51)
Infliximab dose		
5 mg kg^−1^	20 (44)	14 (28)
7.5 mg kg^−1^	5 (11)	12 (23)
10 mg kg^−1^	20 (45)	25 (49)
Infliximab interval		
Q4 weeks	3 (7)	6 (12)
Q6 weeks	11 (24)	11 (21)
Q8 weeks	31 (69)	34 (67)
Type of infliximab received		
Originator	28 (62)	27 (53)
Biosimilar	10 (22)	10 (20)
Both	7 (16)	14 (27)

Data are expressed in *n* (%). Abbreviation: Q = every.

### Infusion Data

Median number of infusions was 7 per patient in the control (IQR 5–8) and study (IQR 6–9) groups, assessed over a median of 12 months in the control and 14 months in the study group (IQR 8–16). Total number of infusions was 309 in the control group, and 376 in the study group. Premedications were used in 57% of infusions in the control group, and 28% of infusions in the study group (χ^2^ = 56.32, *P* < .0001) (Table 3). Diphenhydramine was given in 179 infusions, mostly through the oral route (171/179, 95%).

**Table 3.** Infusion data.

	2-h infusion group (*n* = 45)	1-h infusion group (*n* = 51)
Median number of infusions	7 (5–8)	7 (6–9)
Duration of follow-up (in months)	12 (8–16)	14 (8–16)
Total number of infusions	309	376
Premedications	175 (57)	106 (28)
Acetaminophen	171 (55)	99 (26)
Diphenhydramine	110 (36)	69 (18)
Loratidine	7 (2)	11 (3)
Ondansetron	4 (1)	0 (0)
Methylprednisolone	4 (1)	1 (0.3)

Data are expressed in median (IQR) or *n* (%). Denominator in premedications is total number of infusions in each group. Abbreviation: IQR, interquartile range.

### Infusion Reactions

**Figure 3.**
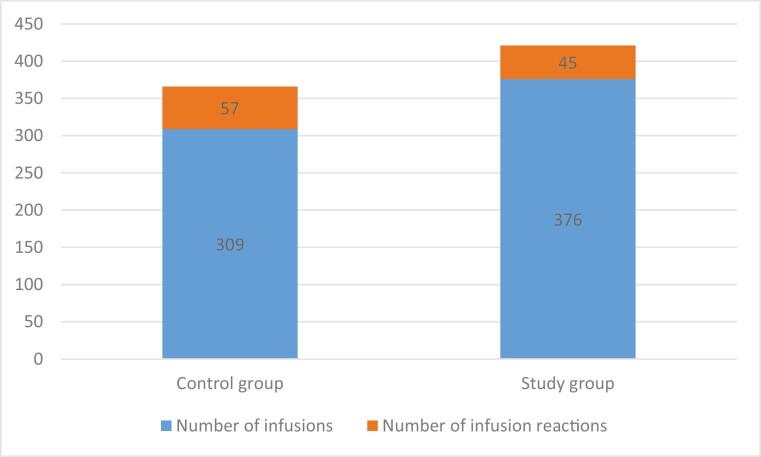
Infusions reactions in control and study groups.

An infusion reaction occurred in 18% of infusions in the control group in 27/45 (60%) of patients, and 12% of infusions in the study group in 30/51 (59%) of patients (Figure 3). The only infusion reaction that occurred in all these patients was hypotension, defined per our protocol as a decrease in baseline systolic blood pressure (SBP) by >20 mm Hg post-infusion compared with pre-infusion, all of which were asymptomatic and none had SBP <85 mm Hg. Based on Farrington–Manning score test for the difference between 2 proportions ([90% CI for the rate difference 0.0165–0.1071], *P* < .0001), the reaction rate for 1-hour infusions was non-inferior to the reaction rate for 2-hour infusions. The association between use of concomitant immunomodulators or premedications and infusion reactions was assessed, and the use of diphenhydramine was associated with an increased rate of infusion reactions, for example, hypotension per protocol (OR 2.04 [95% CI 1.18–3.52], *P* = 0.01). One patient from the study group dropped out of the study after 1 infusion, due to report of fatigue a few days following the infusion.

### Cost Analysis

The standard nursing time spent in our infusion center was 180 minutes for the 2-hour infusion and 105 minutes for the 1-hour infusion. Patients spent an average of 60 minutes driving to and from the infusion center. The average direct nursing cost was $124.41 for the 2-hour infusion and $72.57 for the 1-hour infusion. The average chair occupancy cost at our infusion center was $350 for the 2-hour infusion and $225 for the 1-hour infusion. Including patients’ loss of productivity costs in the analysis, based on the average employee salary in the United States ($11.11 per hour),^34^ the average cost is $507.74 for the 2-hour infusion and $319.79 for the 1-hour infusion (relative reduction −37%). By changing from 2- to 1-hour infusion, the number of patients each chair in the infusion center can treat increases from 3 patients a day to 4 patients a day, a 33% increase.

## Discussion

To our knowledge, this is the first randomized controlled trial comparing the safety and tolerability of standard and accelerated infliximab infusions in IBD patients. Our findings support the non-inferiority of 1-hour infusions compared with 2-hour infusions, with the absence of clinically significant acute infusion reactions in either group.

In our study, infusion reactions were observed in 18% of the standard 2-hour infusions, and 12% in the accelerated 1-hour infusions, similar to those reported in the large randomized controlled trials assessing the efficacy of infliximab.^13^ It is important to note that although hypotension, the only infusion reaction observed in all our patients, was pre-classified as a moderate/severe reaction per our study protocol, no systolic blood pressures dropped below 85 mm Hg, none of these patients were symptomatic, and the change in blood pressure was recorded as part of routine pre-, intra-, and post-infusion monitoring. Moreover, many of these patients experienced normalization of their blood pressures from initial hypertensive readings, a phenomenon well described in primary care clinics, named as “first-pass” white-coat hypertension.^35,36^ There were no infusions discontinued as a result of these reactions.

Other than the per-protocol asymptomatic hypotension, we did not observe any other infusion reactions in either the 1- or 2-hour infusion groups. Infliximab infusion reactions thus appear to be extremely rare once patients demonstrated tolerability of induction therapy.

Almost half of our patients were on concomitant immunomodulators, which have been shown to prevent antibody formation to infliximab.^20^ These antibodies have been implicated in the pathogenesis of infusion reactions, and conflicting evidence has emerged on their role in the prevention of infusion reactions.^14,19,26^ Similar to the meta-analysis reported by Neef et al,^37^ there was no association found between use of concomitant immunomodulators and infusion reactions in our study.

The use of premedications in our study was left to the discretion of the ordering physician and individual patient, and 2-hour infusions were twice as likely to be given with premedications compared with 1-hour infusions. The variability in the prescription of premedications has been reported by Adler et al,^38^ and their utility in the prevention of acute infusion reactions in maintenance infusions has been previously questioned.^18^ In our study, the use of analgesics, anti-emetics, and corticosteroids as premedications had no association with infusion reactions, but the use of first generation antihistamine diphenhydramine, was associated with a 2-fold increased rate of infusion reactions, namely hypotension in our study. This paradoxical increase in infusion reactions with antihistamines has been previously described by Choquette et al.^16^ Although the explanation provided by the previous study for the increased rate was related to selection bias, whereby patients with history of infusion reactions were more like to receive premedication with antihistamines with the hopes of preventing another reaction, this is not applicable in our study since none of our patients had a history of infusion reactions. The most likely explanation in our study relates to diphenhydramine itself, which is known to cause hypotension as a side effect.^39^

Although the 2-hour infusion group was twice as likely to receive premedications as the 1-hour infusion group, the infusion reaction rate of 1-hour infusion is non-inferior to that of 2-hour infusion; in fact, numerically 1-hour infusion had a lower incidence of infusion reactions. This is important to highlight because despite the anticipation that shortening infusion time to 1 hour could potentially increase infusion reactions, the 1-hour infusion group was no more likely to have infusion reactions, even though it was half as likely to receive premedications. This further strengthens our observation that the safety and tolerability of 1-hour infusion is comparable to that of 2-hour infusion.

The average costs per-patient per-infusion in our centers were reduced from $508 in the 2-hour infusion group, to $320 in the 1-hour infusion group, a relative reduction of 37%. This is in agreement with prior studies that have reported a relative cost reduction of up to 51%.^30–32,40,41^ Notably, our study calculated direct nursing costs, indirect infusion center chair occupancy costs and patients’ loss of productivity costs during the infusion.

Between 30% and 60% of IBD patients require infliximab therapy intensification, defined as an increase in infliximab dosage or shortened dosing interval.^42–45^ This is reflected in our study, where 65% of patients required dose escalation, and 32% of patients had a shortened dosing interval. Since we had a similar number of patients with dose intensification in the standard and accelerated infusion groups by means of randomization, this had no bearing on the rate of infusion reactions. Our study adds an important contribution to the mounting evidence supporting the safety of accelerated infliximab infusions, regardless of drug dosing or interval.^24,30,33,37,40^ In addition, we did not observe any difference in infusion reactions between the originator and biosimilar infliximab.

The results of this study should be interpreted in the context of several limitations. First, we excluded patients receiving induction doses of infliximab, patients with a history of infusion reactions and patients with known antidrug antibodies to infliximab, as studies have shown an increased infusion reaction rate in these patients.^16,19,26^ This may have contributed to the absence of clinically significant infusion reactions in our patients. Future studies should assess the safety of accelerated infusions in these groups of patients. Second, we did not assess delayed infusion reactions, as they occur at a much lower rate than acute infusion reactions.^13^ However, we did have 1 patient drop out of the study due to fatigue after an accelerated infusion, which may constitute a delayed infusion reaction.^46^ Finally, the majority of patients assessed for eligibility for the study were excluded primarily due to receiving infusions at outside institutions or patient refusal, and the characteristics of these patients may differ from patients included in the study. However, we did not find any significant differences in their demographics, IBD classification, or infliximab data. In addition, our sample size was sufficiently powered to prove non-inferiority of accelerated infusions.

In summary, our findings demonstrate the safety, tolerability, and cost-effectiveness of accelerated infliximab infusions in patients on maintenance infliximab with no prior infusion reactions. Due to these findings, we propose 1-hour infliximab infusions as the standard of care in IBD patients.

## Supplementary Material

otad022_suppl_Supplementary_Table_S1

## Data Availability

Data not publically available.
