# Supplementary material for: Accelerated Infliximab Infusion Safety and Tolerability Is Non-inferior to Standard Infusion Protocol in Inflammatory Bowel Disease Patients: A Randomized Controlled Study
Source: Crohns Colitis 360. 2023 May 3;5(3):otad022. doi: 10.1093/crocol/otad022 (PMC10243871; doi:10.1093/crocol/otad022)
Supplement: otad022_suppl_Supplementary_Table_S1 [file otad022_suppl_supplementary_table_s1.docx]

**Supplementary Table 1. Comparison between excluded patients (declined and outside infusions) and included patients**

| **Variable** | **p-value** |
| --- | --- |
| **Age** | 0.51 |
| **Legal sex** | 0.83 |
| **IBD classification** | 0.11 |
| **Infliximab dose** | 0.77 |
| **Infliximab interval** | 0.86 |
| **Concomitant immunosuppression** | 0.87 |
